# Maternal and fetal outcomes in pregnant women with liver disease

**DOI:** 10.12669/pjms.42.7.13219

**Published:** 2026-07

**Authors:** Mehnaz Khakwani, Rashida Parveen, Hajra Sultana

**Affiliations:** 1 Mehnaz Khakwani, FCPS, Department of Obstetrics and Gynecology, Nishtar Medical University, Multan, Pakistan; 2 Rashida Parveen, FCPS, Department of Obstetrics and Gynecology, Nishtar Medical University, Multan, Pakistan

**Keywords:** Gestational age, Jaundice, Liver disease, Pregnancy, Vomiting

## Abstract

**Objective:**

To determine the maternal and fetal outcomes among pregnant females with liver disease.

**Methodology:**

This prospective observational study was performed at the department of obstetrics & gynecology, Nishtar Hospital Multan, Pakistan, during August 2024 to July 2025. Pregnant females aged 20-40 years with gestational age ≥ 28 weeks, regardless of their parity or gravidity status, with liver disease, and planned to undergo delivery were analyzed. Demographic, clinical and laboratory parameters were documented. Clinical presentation, causes of liver disease, and details about the treatment were recorded. Fetal and maternal outcomes in terms of survival or death were noted.

**Results:**

Among the 136 women, the median age, gestational age, and BMI were 29.50 (26.00-35.00) years, 36.00 (34.00-38.00) weeks, and 27.65 (25.05-30-98) kg/m^2^, respectively. The most common clinical presentations were jaundice, and vomiting, noted in 31 (22.8%), and 24 (17.6%) women, respectively. The most frequent causes of liver disease was intra-hepatic cholestasis, noted in (25.7%). Maternal mortality was observed in 4 (2.9%) women, and the presence of acute fatty liver disease (AFLD) was more frequent in maternal deaths (p=0.008). There were 5 (3.7%) fetal deaths, and significantly associated with acute fatty liver disease (60.0%, p=0.007).

**Conclusion:**

The findings suggest a possible association of acute fatty liver disease with adverse maternal and fetal outcomes. Although the observed mortality rates were relatively low, careful monitoring and appropriate management of pregnant women with liver disease remain important.

## INTRODUCTION

Abnormal liver function is observed in approximately 3-5% of pregnancies and has the potential to adversely influence maternal and fetal outcomes.^1^,^2^ The physiological adaptations of pregnancy may alter liver function test values, producing distinct patterns that differ from those seen in non-pregnant women.^3^ Among pathological conditions, preeclampsia remains the most frequent cause of liver dysfunction during pregnancy. In some cases, it advances to HELLP (Hemolysis, Elevated Liver enzymes and Low Platelets) syndrome or eclampsia, both of which are associated with serious maternal risks including placental abruption, renal impairment, subcapsular hematoma, and hepatic rupture.^4^-^6^ Another important condition is intrahepatic cholestasis of pregnancy, which characteristically manifests as nocturnal pruritus of the palms and soles and is associated with impaired liver function.^7^ Acute fatty liver disease (AFLD) of pregnancy poses a significant threat to maternal and fetal survival if not rapidly diagnosed and managed.^8^ Liver transplantation is the treatment of choice in end-stage liver disease exhibiting improved post-transplant survival enabling women to conceive successfully.^9^,^10^

Evidence from local research indicated fulminant hepatic failure (FHF) as he commonest cause behind liver disease in pregnancy (LDP), affecting 14.9% women, with high maternal (11.6%) and neonatal (39.6%) mortality.^11^ These findings emphasize the serious consequences of LDP. However, despite its clinical importance, there is limited evidence from Pakistan addressing maternal and fetal outcomes in this population. Therefore, the present study was designed to determine maternal and fetal outcomes among pregnant women with liver disease.

## METHODOLOGY

This prospective observational study was conducted in the Department of Obstetrics and Gynecology, Nishtar Hospital Multan, Pakistan, over a one-year period from August 2024 to July 2025. . The study population consisted of pregnant women aged 20 to 40 years with a gestational age of 28 weeks or more who had a confirmed diagnosis of liver disease and were planned for delivery. Liver disease was operationally defined as the presence of at least one abnormal hepatic biochemical parameter, including ALT >40 IU/L, AST >40 IU/L, total bilirubin >1.2 mg/dL, or prolonged prothrombin time >14 seconds/INR >1.2, in association with a clinically identifiable hepatic disorder during pregnancy. Women with pre-existing renal disease or any type of malignancy were excluded. A sample size of 136 was calculated based on the reported prevalence of FHF as 14.9% in pregnancy,^11^ with a 95% confidence level and a 6% margin of error. Eligible women were recruited consecutively.

### Ethical Approval:

It was obtained from the Institutional Ethical Committee (Letter No: 12513/NMU, dated 7-08-2024), and written informed consent was secured from all participants before enrollment

Baseline demographic and clinical characteristics of all enrolled women were recorded. Relevant laboratory investigations were performed according to the suspected clinical diagnosis and included complete blood count with platelet count, liver function tests including serum bilirubin, ALT, AST, alkaline phosphatase (ALP) and serum albumin, coagulation profile including prothrombin time and international normalized ratio (INR), renal function tests, blood glucose, serum bile acids where intrahepatic cholestasis of pregnancy was suspected, and viral hepatitis markers including HBsAg, anti-HCV, HAV IgM and HEV IgM where indicated. These investigations were used for diagnostic classification and clinical management. Clinical presentation, cause of liver disease, treatment details, and both maternal and fetal outcomes were documented on a structured proforma. Preeclampsia was defined as new-onset hypertension after 20 weeks of gestation with proteinuria of ≥300 mg/24 hours or evidence of end-organ dysfunction. Eclampsia was defined as preeclampsia complicated by generalized tonic-clonic seizures not attributable to other causes. HELLP syndrome was identified by the triad of hemolysis, elevated liver enzymes (AST/ALT ≥70 IU/L), and a platelet count of ≤100,000/µL. AFLD was diagnosed clinically and supported by laboratory evidence including hypoglycemia, elevated transaminases, and coagulopathy, with reference to Swansea criteria.^12^ Intrahepatic cholestasis (IHC) of pregnancy was defined as the presence of pruritus with elevated serum bile acids (>10 µmol/L) and/or raised transaminases in the absence of other liver disease. Maternal outcome was categorized as survival or death during hospitalization, while fetal outcome was classified as survival (live birth) or death (stillbirth or neonatal death before discharge).

Clinical information and laboratory results were obtained at and during the admission, while maternal and fetal outcomes were assessed following delivery. Data analysis was performed using IBM SPSS Statistics version 26.0. Quantitative variables were presented as mean±standard deviation (SD), or median and interquartile range (IQR), whereas categorical variables were summarized as frequencies and percentages. Chi-square tests was used to compare maternal and fetal outcomes with respect to study variables, taking p<0.05 as statistically significant.

## RESULTS

Among the 136 women, the median age, gestational age, and BMI were 29.5 (26.0-35.0) years, 36.0 (34.0-38.0) weeks, and 27.7 (25.05-30.9) kg/m^2^, respectively. There were 90 (66.2%) women who belonged to rural areas, while 46 (33.8%) were from urban areas. The distribution of gestational age showed that 56.6% were preterm. The maternal education level indicated that 46 (33.8%) women were illiterate. The prevalence of comorbidities included gestational diabetes mellitus, and hypertension, in 42 (30.9%), and 32 (23.5%) women, respectively. The most common clinical presentations were jaundice, and vomiting, noted in 31 (22.8%), and 24 (17.6%) women, respectively. The most frequent cause of liver disease was IHC, noted in (25.7%) (Table-I).

**Table-I:** Demographic and clinical characteristics (n=136).

Characteristics		Number (%)
Age (years)	20-30	75 (55.1%)
	31-40	61 (44.9%)
Residence	Rural	90 (66.2%)
	Urban	46 (33.8%)
Gestational age	Pre-term	77 (56.6%)
	Term	59 (43.4%)
BMI (kg/m^2^)	<25	31(22.8%)
	≥25	105 (77.2%)
Parity	Nulliparous	64 (47.1%)
	Multiparous	72 (52.9%)
Maternal education	Illiterate	46 (33.8%)
	Literate	90 (66.2%)
Diabetes mellitus/Gestational diabetes mellitus		42 (30.9%)
Hypertension		32 (23.5%)
Clinical presentation frequency	Abdominal pain	21 (15.4%)
	Altered level of consciousness	21 (15.4%)
	Jaundice	31 (22.8%)
	Nausea	20 (14.7%)
	Pruritus	19 (14.0%)
	Vomiting	24 (17.6%)
Causes of liver disease	Acute fatty liver disease	4 (2.9%)
	HELLP	7 (5.1%)
	Intra-hepatic cholestasis	35 (25.7%)
	Pre-eclampsia	24 (17.6%)
Mode of delivery	Vaginal delivery	62 (45.6%)
	Cesarean section	74 (54.4%)

The median ALT, AST, ALP, serum albumin, prothrombin time, INR, and activated partial thromboplastin time (aPTT) were 49.0 U/L (39.0-60.0), 51.0 U/L (37.0-58.0), 129.0 U/L (114.3-139.8), 3.0 g/dL (2.8-3.2), 14.5 seconds, (13.2-15.9), 1.3 (1.2-1.4), and 35.5 seconds (32.0-38.0), respectively. The median hemoglobin, creatinine, and urea levels were 12.00 g/dL, (10.0-12.0), 1.0 mg/dL (0.9-1.2) and 24.0 mg/dL (22.0-27.0), respectively.

Of the 136 women, 132 (97.1%) survived, while 4 (2.9%) died. Maternal mortality was not significantly associated with age (p=0.418), residence (p=0.705), gestational age (p=0.195), or BMI (p=0.188). Maternal mortality was relatively higher among multiparous women (p=0.056). The presence of AFLD was strongly associated with maternal death (p=0.008). None of the clinical presentations were having any significant association with maternal mortality (p>0.05) (Table-II).

**Table-II:** Association of maternal mortality with characteristics of women (n=136).

Characteristics		Final outcome		P-value
		Survived (n=132)	Mortality (n=4)	
Age (years)	20-30	72 (54.5%)	3 (75.0%)	0.418
	31-40	60 (45.5%)	1 (25.0%)	
Residence	Rural	87 (65.9%)	3 (75.0%)	0.705
	Urban	45 (34.1%)	1 (25.0%)	
Gestational age	Pre-term	76 (57.6%)	1 (25.0%)	0.195
	Term	56 (42.4%)	3 (75.0%)	
BMI (kg/m^2^)	<25	29 (22.0%)	2 (50.0%)	0.188
	≥25	103 (78.0%)	2 (50.0%)	
Parity	Nulliparous	64 (48.5%)	-	0.056
	Multiparous	68 (51.5%)	4 (100%)	
Maternal education	Illiterate	46 (34.8%)	-	0.147
	Literate	86 (65.2%)	4 (100%)	
Diabetes mellitus/Gestational diabetes mellitus		42 (31.8%)	-	0.179
Hypertension		31 (23.5%)	1 (25.0%)	0.944
Clinical presentation frequency	Abdominal pain	21 (15.9%)	-	0.386
	Altered level of consciousness	20 (15.2%)	1 (25.0%)	0.591
	Jaundice	29 (22.0%)	2 (50.0%)	0.886
	Nausea	20 (15.2%)	-	0.399
	Pruritus	19 (14.4%)	-	0.413
	Vomiting	23 (17.4%)	1 (25.0%)	0.695
Causes of liver disease	Acute fatty liver disease	3 (2.3%)	1 (25.0%)	0.008
	HELLP	7 (5.3%)	-	0.636
	Intra-hepatic cholestasis	35 (26.5%)	-	0.232
	Pre-eclampsia	24 (18.2%)	-	0.347

P-values calculated using Fisher’s exact test where appropriate.

There were 5 (3.7%) fetal deaths, while AFLD was found to have significant association with fetal mortality (p=0.021). Fetal mortality was not significantly associated with maternal age (p=0.488), residence (p=0.766), gestational age (p=0.445), or BMI (p=0.879). While there was no significant association between parity and fetal death (p=0.133), although higher proportion of fetal deaths occurred among multiparous women (80.0%). Clinical presentations did not significantly correlate with fetal mortality (p>0.05). Table-III presents the association between maternal characteristics and fetal mortality.

**Table-III:** Association of fetal mortality with characteristics of women (n=136).

Characteristics		Fetal death		P-value
		No (n=131)	Yes (n=5)	
Age (years)	20-30	73 (55.7%)	2 (40.0%)	0.488
	31-40	58 (44.3%)	3 (60.0%)	
Residence	Rural	87 (66.4%)	3 (60.0%)	0.766
	Urban	44 (33.6%)	2 (40.0%)	
Gestational age	Pre-term	75 (57.3%)	2 (40.0%)	0.445
	Term	56 (42.7%)	3 (60.0%)	
BMI (kg/m^2^)	<25	30 (22.9%)	1 (20.0%)	0.879
	≥25	101 (77.1%)	4 (80.0%)	
Parity	Nulliparous	71 (54.2%)	1 (20.0%)	0.133
	Multiparous	60 (45.8%)	4 (80.0%)	
Maternal education	Illiterate	43 (32.8%)	3 (60.0%)	0.207
	Literate	88 (67.2%)	2 (40.0%)
Diabetes mellitus/Gestational diabetes mellitus		39 (29.8%)	3 (60.0%)	0.151
Hypertension		32 (24.4%)	-	0.206
Clinical presentation frequency	Abdominal pain	20 (15.3%)	1 (20.0%)	0.774
	Altered level of consciousness	20 (15.3%)	1 (20.0%)	0.774
	Jaundice	30 (22.9%)	1 (20.0%)	0.879
	Nausea	19 (14.5%)	1 (20.0%)	0.733
	Pruritus	18 (13.7%)	1 (20.0%)	0.692
	Vomiting	24 (18.3%)	-	0.292
Causes of liver disease	Acute fatty liver disease	3 (2.3%)	1 (20.0%)	0.021
	HELLP	7 (5.3%)	-	0.596
	Intra-hepatic cholestasis	35 (26.7%)	-	0.180
	Pre-eclampsia	22 (16.8%)	2 (40.0%)	0.182

P-values calculated using Fisher’s exact test where appropriate.

## DISCUSSION

The maternal mortality rate in this study was 2.9%. Farooq et al.,^13^ reported higher maternal mortality rates of 13.3%, primarily associated with complications from Hepatitis E and fulminant hepatic failure (FHF). This disparity in mortality rates could be attributed to differences in healthcare access, quality of care, and the timing of diagnosis and intervention in different regions. In this study, AFLD showed a significant association with maternal mortality (p=0.008). Tolunay et al.,^14^ reported that hepatic cirrhosis and severe liver dysfunction were major contributors to maternal death. The fetal mortality rate observed here was 3.7%, which is lower than the 13.5% reported by Panchbudhe et al.,^15^ where conditions such as Hepatitis E and HELLP syndrome were implicated. Fetal mortality in the present study was also significantly linked to AFLD (p=0.021). Gao et al.,^16^ identified liver cirrhosis in pregnancy as a key risk factor for fetal death and preterm birth. The comparatively lower fetal mortality rate in this study may reflect improved management strategies.

The most common clinical presentations in this study were jaundice (22.8%), and vomiting (17.6%), both of which are classic signs of liver dysfunction.^6^ These symptoms, along with pruritus and abdominal pain, are frequently observed in pregnancy-related liver diseases. Butt et al.,^11^ identified acute hepatitis E and preeclampsia as the most common causes of liver disease during pregnancy, and with those of Tiwari et al.,^17^ where hypertensive disorders of pregnancy were identified as the most frequent causes of liver dysfunction in pregnancy.

The prevalence of IHC in this research was relatively higher than Farooq et al.,^13^ where the prevalence of ICP was lower, possibly reflecting regional differences in etiological factors and genetic predispositions. The prevalence of AFLD in this study (2.9%) is noteworthy, especially considering it to be more frequent among women who died. Rasheed et al.,^18^ highlighted the severity of AFLD which can lead to fulminant hepatic failure if not identified and managed promptly. Global evidence suggests that AFLD of pregnancy is one of the most dangerous liver conditions during pregnancy, with a significantly increased risk of mortality.^19^,^20^

The study documented a trend towards increased elevated liver enzymes, suggest a moderate degree of liver dysfunction among pregnant women with liver disease. These findings are consistent with Kumar et al.,^21^ where liver enzyme levels were similarly elevated, indicating hepatic injury and impaired liver function. The reduced serum albumin levels (median: 3.00 g/dL) and prolonged prothrombin time highlight the compromised synthetic function of the liver, which is a hallmark of severe liver dysfunction in pregnancy.^22^

In this study, cesarean section emerged as the predominant mode of delivery, accounting for 54.4% of cases. This finding is consistent with Tiwari et al.,^17^ and Tan et al.,^23^ who also reported a high rate of cesarean deliveries in pregnancies complicated by liver disease. Gao et al.,^16^ observed that cesarean delivery was more frequent among women with liver cirrhosis, largely due to complications such as hemorrhage and fetal distress. The elevated cesarean rate in the present cohort likely reflects the urgent need for delivery in cases of maternal liver failure or fetal compromise, both of which are common in severe liver disease.^24^

The strong link between AFLD and maternal mortality highlights the importance of identifying at-risk pregnancies early and initiating timely interventions. Because the clinical manifestations of LDP can mimic other common conditions such as preeclampsia, clinicians must maintain a high level of vigilance when evaluating women with jaundice, vomiting, or abdominal pain.^25^ The high proportion of cesarean deliveries in this study further emphasizes the need for close monitoring and rapid decision-making regarding delivery. In resource-limited settings, effective collaboration between obstetricians and hepatologists is essential to ensure timely delivery and optimal management of potential complications.

### Limitations

The sample size was relatively small, and the number of adverse maternal and fetal events was low, which may have limited the statistical power to detect significant associations. As this was a single-center hospital-based study conducted in a tertiary care setting, potential selection bias cannot be excluded as more complicated cases may have been referred or admitted. Due to the low event rate, multivariable regression analysis could not be performed reliably, therefore, the observed associations should be interpreted as unadjusted findings. The absence of follow-up data restricts the evaluation of long-term outcomes. The study nature precludes establishing causal relationships.

## CONCLUSION

The findings suggest a possible association of acute fatty liver disease with adverse maternal and fetal outcomes. Although the observed mortality rates were relatively low, careful monitoring and appropriate management of pregnant women with liver disease remain important.

### Authors contribution:

**MK:** Literature search**,** Data collection and synthesis, critical review.

**RP:** Data synthesis, drafting, data analysis,

Both the authors are responsible for integrity of the study and have also approved the final version of the manuscript.
